# Pancreatic Pseudocyst Presenting as Dysphagia: A Case Report

**DOI:** 10.4103/1319-3767.37801

**Published:** 2008-01

**Authors:** Charles Panackel, Arun T. Korah, Devadas Krishnadas, Kattoor R. Vinayakumar

**Affiliations:** Department of Medical Gastroenterology, Medical College, Trivandrum, Kerala, India

**Keywords:** Pancreatic pseudocyst, dysphagia, mediastinal cyst, alcoholic pancreatitis

## Abstract

Pancreatic pseudocysts are relatively common complications of acute pancreatitis. However, extension of pseudocysts into the mediastinum rarely occurs. In such situations they commonly present with chest pain or shortness of breath. We herein report the case of a patient with a pseudocyst presenting with dysphagia. The clinical presentation, current modalities of diagnosis and management of mediastinal pancreatic pseudocyst is reviewed in this article.

Mediastinal pseudocyst is a rare complication of pancreatitis. A mediastinal pseudocyst occurs when the pancreatic duct ruptures posteriorly into retroperitoneum and the fluid tracks into mediastinum.[1] In the majority of patients, the pancreatic fluid enters the mediastinum through the esophageal or aortic hiatus. Other less frequent sites of entry into the mediastinum are the foramen of Morgagni, the inferior vena cava hiatus and direct penetration of the diaphragm. The symptoms in mediastinal pseudocysts are nonspecific.[2] Abdominal symptoms may be absent as the pseudocyst can easily decompress into the low-pressure thoracic cavity. Rarely, the patients may present with dysphagia, odynophagia, dyspnoea and congestive heart failure. We present a case of mediastinal pseudocyst presenting as dysphagia.

## CASE REPORT

A 35-year-old male was admitted with progressive dysphagia of three weeks duration. He had dysphagia for both solids and liquids. There was no fever or odynophagia. Patient gave no history of ingestion of corrosives, pills or foreign bodies. He did not have cough, dyspnoea orthopnea or paroxysmal nocturnal dyspnoea. There was no history of vomiting, hemoptysis or hematemesis and weight loss. He gave a history of severe upper abdominal pain two months back. Pain occurred following a binge of alcohol and lasted for two days. Pain was relieved by symptomatic treatment from a local hospital. Patient did not have any further episodes of abdominal pain.

**Figure 1:**
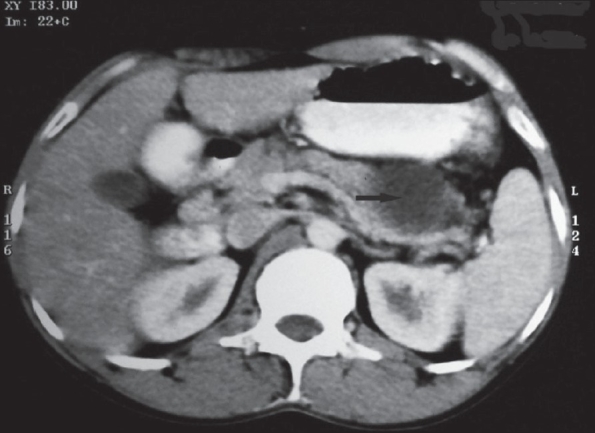
CT scan abdomen after oral and IV contrast showing a cystic lesion in the region of tail of pancreas suggestive of pancreatic pseudocyst

**Figure 2:**
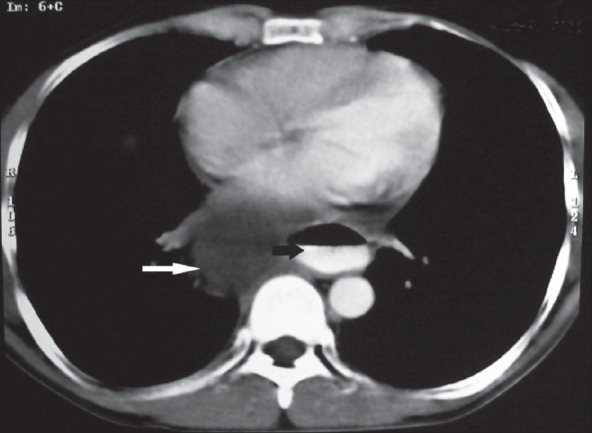
CT scan of lower chest after oral and IV contrast showing a cystic lesion in posterior mediastinum compressing the esophagus. Air contrast level is seen in lower esophagus suggestive of stasis

On clinical examination, the patient was found to be afebrile and the vital signs were stable. He was well built and nourished. There was no pallor, cyanosis, clubbing, jaundice and lymphadenopathy. There was no oral ulceration or candidiasis. There were no stigmata of internal malignancy. Chest examination was normal. Abdominal examination did not reveal any evidence of ascites or pancreatitis. There was no evidence of cranial nerve palsy, proximal muscle weakness or ocular defect. Investigations revealed a hemoglobin level of 13.5 gm%, total leukocyte count of 9800 cells/mm^3^ and an erythrocyte sedimentation rate of 15 mm/hr. Liver function and renal function tests were normal. Chest X-ray and electrocardiogram were normal. Esophagogastroscopy was performed, which showed stasis at the lower end of esophagus with an extrinsic compression of the lower esophagus. Ultrasound abdomen showed a large cystic lesion in the upper abdomen in close relation to the tail region of the pancreas. Rest of the abdomen was normal. The values for serum amylase and lipase were 734 U/L and 945 U/L, respectively. With these findings, a computed tomography (CT) scan of abdomen and chest was obtain after administering oral and IV contrast [Figures 1-2]. Computed tomography scan revealed a pancreatic pseudocyst arising in the tail region of the pancreas and extending through the diaphragmatic hiatus into posterior mediastinum. The pseudocyst compressed the esophagus and produced dysphagia.

## DISCUSSION

A pancreatic pseudocyst is defined as a collection of pancreatic juice enclosed by a nonepithelialized wall that occurs as a result of acute pancreatitis, pancreatic trauma or chronic pancreatitis. Pseudocysts are said to occur in 16–50% of cases of acute pancreatitis and in 20–40% of cases of chronic pancreatitis. Seventy to eighty percent of pancreatic pseudocysts are related to alcoholic pancreatitis.[1] We present a case of acute alcoholic pancreatitis with mediastinal pseudocyst as complication. Literature search revealed that the mediastinal pseudocyst of pancreas was more common in men and in those with alcoholic pancreatitis. The most common presenting symptoms of pancreatic pseudocyst were chest or abdominal pains and dyspnoea. Dysphagia was a rare presenting symptom. Most of the patients were treated either by surgical or endoscopic drainage of the cyst. Our patient was treated with octreotide and parenteral nutrition with complete resolution of cyst. This is in line with previous case reports.[3–7]

In acute pancreatitis, the pseudocyst represents the inflammatory response to tissue injury resulting in the formation of fibrous or granulation tissue surrounding an inflammatory exudate of fluid. However, in chronic pancreatitis leads to duct rupture and pseudocyst with subsequent disruption of the pancreatic duct due to increased intraductal pressure.[2]

Pancreatic pseudocysts are typically formed and confined to the lesser omental sac. Mediastinal pseudocyst is a rare thoracic complication of pancreatitis. A cystic posterior mediastinal mass that develops over a short time in a patient with the evidence of pancreatitis is likely to be a pseudocyst. A mediastinal pseudocyst occurs when the pancreatic duct ruptures posteriorly into retroperitoneum and the fluid tracks into mediastinum. In the majority of patients, the pancreatic fluid enters the mediastinum through the esophageal or aortic hiatus. Other less frequent sites of entry into the mediastinum are the foramen of Morgagni, the inferior vena cava hiatus and direct penetration of the diaphragm. In the present case, the posterior mediastinal pseudocyst tracked through the esophageal hiatus. The symptoms in mediastinal pseudocysts are nonspecific. Abdominal symptoms may be absent as the pseudocyst can easily decompress into the low-pressure thoracic cavity. Rarely, the patients may present with dysphagia, odynophagia, dyspnoea and congestive heart failure.[1,2,8,9]

Ultrasound is a quick, inexpensive radiological investigation for diagnosing intraabdominal pseudocysts. However, it cannot determine the presence of mediastinal extension. Computerized tomography (CT) or magnetic resonance imaging (MRI) defines the location and extent of mediastinal pseudocysts. Computerized tomography shows a thin, cystic, low-attenuation mass in the mediastinum or adjacent thoracic cavity.[8] Cyst contents can be isoattenuating or hyperattenuating in relation to water, depending on the presence of hemorrhage or infection. MR imaging demonstrates the cystic nature of the mass and assists in delineating the communication of mediastinal pseudocysts with an abdominal pseudocyst.[10] Newer techniques such as endoscopic ultrasound (EUS) and EUS-guided aspiration of fluid from a mediastinal cyst with an elevated amylase level can confirm the diagnosis of a mediastinal pseudocyst.[11]

The ideal management of mediastinal pseudocysts is controversial and depends on the underlying ductal anatomy, size of the pseudocyst, presence of complications and available expertise. The treatment options include conservative medical management, internal and external drainage of cyst and surgery. Cyst drainage can be radiologically guided external drainage, endoscopic drainage and surgical external or internal drainage. Radiologically guided external drainage has low morbidity and mortality. However, it is complicated by infection, fistula and recurrence. In the past, surgical treatment was the most common therapeutic modality for patients with mediastinal pseudocysts.[12] At present, surgery is considered in symptomatic patients if there is an associated complication such as infection, obstruction, rupture or hemorrhage. Endoscopic retrograde cholangiopancreatography (ERCP) and endoscopic ultrasound (EUS) are potential techniques for the management of mediastinal pseudocyst. Endoscopic therapeutic options include ERCP and transpapillary drainage with or without stent placement for a communicating pseudocyst.[13] EUS guided transesophageal and transhiatal drainage of mediastinal pseudocyst has a high success rate with low mortality and morbidity.[14,15] In the present case, the patient was treated with octreotide 200 *µ*g subcutaneously for 10 days along with intravenous fluids and a complete resolution of cyst was attained.

In conclusion, mediastinal pseudocyst is a very rare complication of pancreatitis. Conservative treatment can be attempted with success in stable patients who have no cardiac or pulmonary compromise.
